# A high-throughput *in vitro* ring assay for vasoactivity using magnetic 3D bioprinting

**DOI:** 10.1038/srep30640

**Published:** 2016-08-01

**Authors:** Hubert Tseng, Jacob A. Gage, William L. Haisler, Shane K. Neeley, Tsaiwei Shen, Chris Hebel, Herbert G. Barthlow, Matthew Wagoner, Glauco R. Souza

**Affiliations:** 1 Nano3D Biosciences, Houston, TX 77030, USA; 2 LC Sciences, Houston, TX 77030, USA; 3 AstraZeneca, Waltham, MA 02451, USA

## Abstract

Vasoactive liabilities are typically assayed using wire myography, which is limited by its high cost and low throughput. To meet the demand for higher throughput *in vitro* alternatives, this study introduces a magnetic 3D bioprinting-based vasoactivity assay. The principle behind this assay is the magnetic printing of vascular smooth muscle cells into 3D rings that functionally represent blood vessel segments, whose contraction can be altered by vasodilators and vasoconstrictors. A cost-effective imaging modality employing a mobile device is used to capture contraction with high throughput. The goal of this study was to validate ring contraction as a measure of vasoactivity, using a small panel of known vasoactive drugs. *In vitro* responses of the rings matched outcomes predicted by *in vivo* pharmacology, and were supported by immunohistochemistry. Altogether, this ring assay robustly models vasoactivity, which could meet the need for higher throughput *in vitro* alternatives.

Cardiovascular liabilities are some of the most prominent reasons for post-approval drug withdrawal^1^,^2^,^3^,^4^. However, the options for early screening of vasoactivity are limited given the paucity of adequate assays currently available. Wire myography is the standard method of evaluating the contractile forces and corresponding geometric changes of an *ex vivo* blood vessel obtained from either animals or human cadaver tissue in response to a compound at varying dosages^5^,^6^,^7^,^8^,^9^. While this method can reliably predict *in vivo* vasoactive responses, high cost and low throughput limit its potential as an early screen for such liabilities. Moreover, the limited relevance of non-human tissues and the need to reduce the dependence on animal-intensive tests like wire myography define an unmet need for *in vitro* assays with fewer ethical challenges and the potential for greater translational capabilities. Thus, there is a demand for an *in vitro* assay that is predictive of *in vivo* vasoactive responses, uses human cells, and is adaptable to high-throughput screening, as either an alternative to wire myography or earlier screen for vasoactivity before wire myography.

One of the many reasons for the lack of adequate *in vitro* assays is the environment in which these assays are typically run. Rigid two-dimensional (2D) plastic or glass surfaces poorly represent natively compliant vasculature^10^,^11^,^12^,^13^,^14^. Cells used in these assays are also uniformly exposed to biochemical factors in the media, whereas in native tissue, cells are exposed to a gradient of compounds^10^. Furthermore, the extracellular matrix (ECM) in native tissue regulates compound diffusion and cell signaling via cell-ECM interactions, which are difficult to replicate in 2D^11^,^12^,^13^,^14^. Thus, any *in vitro* assay for vasoactivity must capture the three-dimensional (3D) environment of the native vessel to recreate smooth muscle contractility.

To overcome these challenges, this study introduces a novel assay for vasoactivity using magnetic 3D bioprinting. Magnetic 3D bioprinting is a method to engineer tissues by magnetizing and printing cells using magnetic forces. Magnetization is accomplished by incubation with a biocompatible nanoparticle assembly consisting of gold, iron oxide, and poly-L-lysine^15^,^16^,^17^,^18^,^19^,^20^,^21^,^22^,^23^,^24^,^25^,^26^,^27^. Once magnetized, these cells can be rapidly printed with high reproducibility using mild magnetic forces. To study vasoactivity, this method prints vascular smooth muscle cells into 3D rings that structurally represent blood vessel segments. Importantly, these rings contract immediately and spontaneously after printing and vary with compound concentration. Similar assays have been developed for wound healing in rings^24^, and toxicity in spheroids^27^. Assessment of functional responses is accomplished with a mobile device-based imaging system, made possible by the computing power of mobile devices today, and contrast between the dark magnetized ring of cells and the media. This imaging system allows for high-throughput screening by automating the imaging of whole plates of rings at regular intervals (≥1 s), thereby increasing throughput and offering kinetic analysis compared to traditional well-by-well imaging under a microscope^24^,^27^. Taken together, this assay has the potential to meet the needs for an *in vitro* assay for high-throughput vasoactivity screening.

The goal of this study was to validate this ring assay as an *in vitro* measure of vasoactivity. Rings were printed using A10 rat vascular smooth muscle cells and primary human aortic smooth muscle cells (ASMC) to demonstrate the ability to assay human cells, a limitation of wire myography. The rings were exposed to a small panel of compounds with known vasoactive responses (vasodilators - blebbistatin, forskolin, verapamil; vasoconstrictors - norepinephrine, phenylephrine, U46619). The contractile responses of rings were measured over 24 h of exposure, as well as their viability. Their expression of α-smooth muscle actin (αSMA), a cytoskeletal component and smooth muscle marker^28^, and phosphorylated myosin light chain (pMLC), a precursor for vascular smooth muscle contraction were analyzed with immunohistochemistry^29^. The results of this study validate contraction in magnetically 3D bioprinted rings as an endpoint for vasoactivity, and form the foundation for high-throughput screening of vasoactivity.

## Results

### Ring Contraction

**Figure 1:**
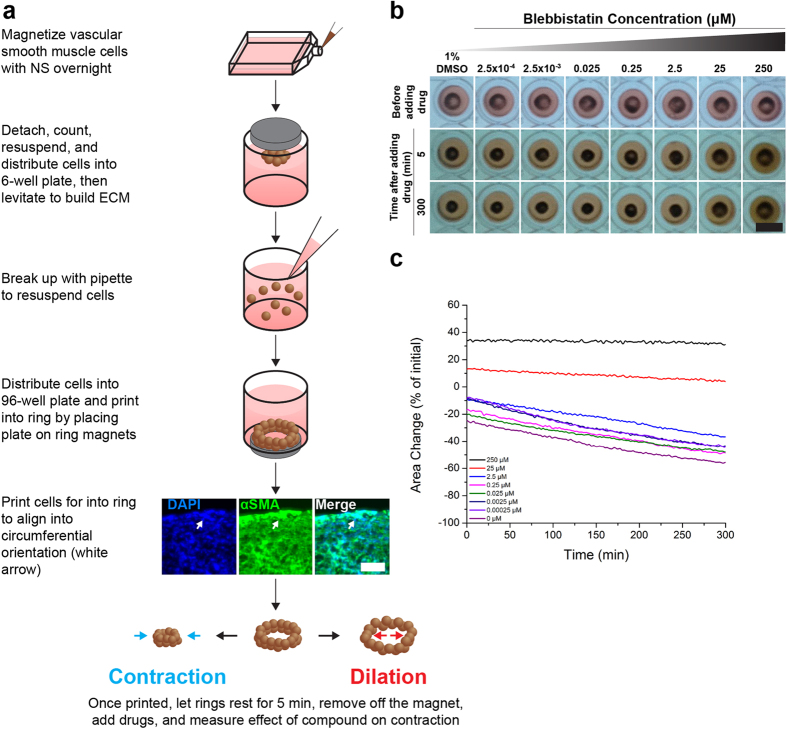
Magnetic 3D bioprinting vascular smooth muscle cells. (**a**) Cells were first magnetized overnight with NanoShuttle. The next day, cells were detached, counted, resuspended, and distributed into a 6-well plate to levitate for 2 h to build ECM. The cells were then resuspended, and distributed into a 96-well plate. The plate was placed atop a magnetic drive of 96 ring magnets that print the cells into a 3D ring. Printing helps align the cells into a circumferential orientation within the ring, as suggested by the alignment of αSMA. After printing, drugs were added to each well and contraction was imaged and measured. (**b**) Representative images of A10 rings taken with the mobile device-based imaging system with varying concentrations of blebbistatin, which was repeated 3 times (n = 3 rings for 8 concentrations of each compound). (**c**) Area change seen in rings measured from images over time. Note that the rings naturally contract, and that blebbistatin has a dilatory effect on ring contraction at higher concentrations. White scale bar = 50 μm. Black scale bar = 5 mm.

**Figure 2:**
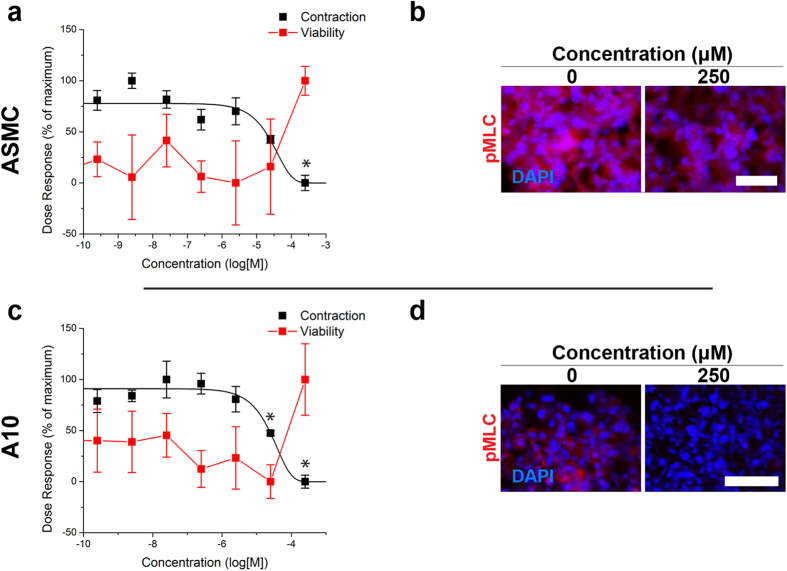
Effects of blebbistatin on vascular smooth muscle ring contraction. The effects of blebbistatin on ring contraction after 1 h (black) and viability (red) in ASMC (**a**) and A10 (**c**) rings showed an expected result, in which ASMC and A10 rings were unable to contract as quickly as vehicle-treated rings with higher concentrations of blebbistatin, without any toxic effect (n = 3 rings for 8 concentrations). Immunofluorescent stains of ASMC (**b**) and A10 (**d**) rings for pMLC (red) of those rings after 5 min of contraction, with nuclei counterstained with DAPI (blue), showed that with higher amounts of blebbistatin, which binds myosin light chain, the rings stain less intensely for pMLC, in keeping with the slower rate of contraction. 100% = maximum, 0% = minimum. Scale bar = 50 μm. *p < 0.05 v. control.

**Figure 3:**
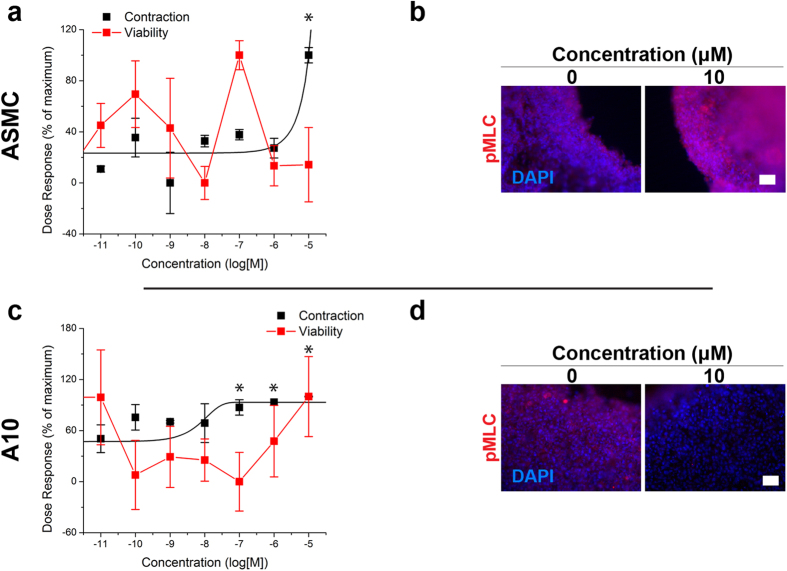
Effects of norepinephrine on vascular smooth muscle ring contraction. Norepinphrine had an expected effect on ring contraction (black) and viability (red) in ASMC (**a**, contraction after 1.5 h) and A10 (**c**, contraction over 4 h), where ASMC and A10 rings contracted faster than controls without any toxic effect (n = 3 rings for 8 concentrations). Immunofluorescent stains of ASMC (**b**) and A10 (**d**) rings for pMLC (red) of those rings after 5 min of contraction, with nuclei counterstained with DAPI (blue), showed that the stain intensity for pMLC increased in ASMC rings with higher concentrations of norepinephrine, but decreased in A10 rings. 100% = maximum, 0% = minimum. Scale bar = 50 μm. *p < 0.05 v. control.

**Figure 4:**
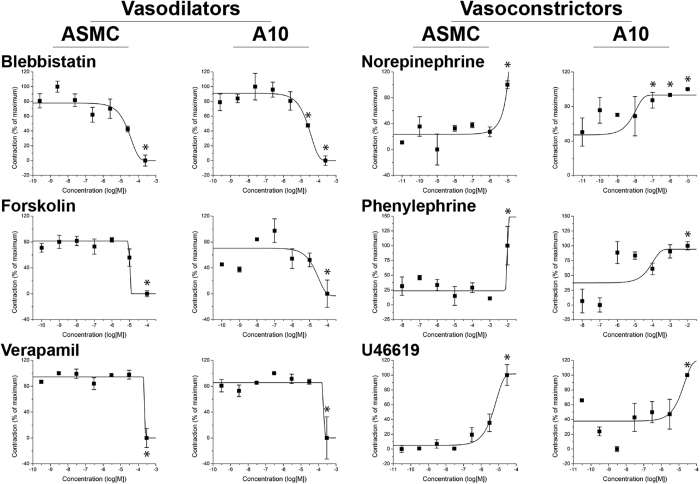
Dose-response of vascular smooth muscle rings. Contraction of ASMC and A10 rings as a function of compound concentration (n = 3 rings for 8 concentrations of each compound). Note that in general, vasodilators slowed smooth muscle contraction, while vasoconstrictors increased contraction with higher doses. 100% = maximum contraction, 0% = minimum contraction, *p < 0.05 v. control.

ASMCs and A10s were successfully printed into rings that contracted immediately after printing (Fig. 1, see Supplemental Fig. SF1 for timeseries of rings in response to all compounds). To assess the contractile capability of the microtissues, their projected areas were tracked during exposure to three vasodilators and three vasoconstrictors for up to 5 hours (Table 1, see Supplemental Table ST1 for endpoints). Vasodilators, such as blebbistatin, had a significant dose-dependent response representative of dilation, in which higher concentrations reduced contraction, in both ASMC and A10 rings, without reducing viability (Fig. 2a,c). Vasoconstrictors, such as norepinephrine, exhibited a significant contractile effect by increasing ring contraction at higher concentrations in both ASMC and A10 rings, also without reducing viability (Fig. 3a,c). Other vasodilators and vasoconstrictors followed similar trends in contraction, in a significantly concentration-dependent manner (p < 0.005, Fig. 4, Supplemental Tables ST2–4 for p-values). At the same time, there was no significant effect of compound concentration on viability with a significantly toxic effect found only in ASMCs exposed to U46619 (p < 0.05, see Supplemental Fig. SF2 for viability graphs, Supplemental Table ST5 for p-values).

### Immunohistochemistry

ASMC and A10 rings were immunohistochemically stained for pMLC and αSMA. When exposed to vasodilators, like blebbistatin, the stain intensity for pMLC in ASMC rings was slightly lower at higher concentrations compared to control (Fig. 2b, see Supplemental Fig. SF3 for other compounds), while it was notably lower in A10 rings (Fig. 2d, see Supplemental Fig. SF4 for other compounds). Vasoconstrictors, like norepinephrine, had a higher stain intensity for pMLC in ASMC rings at the highest concentration (10 μM norepinephrine, Fig. 3b), while it did not appear to vary in A10 rings with increasing concentrations (Fig. 3d). When stained for αSMA, rings of both cell types stained positively with all compounds and concentrations (for immunofluorescence stains for αSMA and negative controls with all compounds and concentrations for both cell types, see Supplemental Fig. SF5–8). Confocal microscopy of an ASMC ring exposed to 1% DMSO control showed a circumferential orientation of αSMA alignment within the ring, particularly at the edges of the rings (Fig. 1a, see Supplemental Fig. SF9 for negative control).

## Discussion

Contraction of vascular smooth muscle tissue is a key measure of vasoactivity, and has long been used as a surrogate for *in vivo* assessment of vasodilation and vasoconstriction. Here, we describe a novel vasoactivity assay for high-throughput *in vitro* assessment. Magnetic 3D bioprinting was used to print 3D rings of vascular smooth muscle cells that structurally and functionally mimic blood vessel segments by altering the dynamics of contraction in response to a diverse set of vasodilators and vasoconstrictors. This has been achieved using both primary human aortic smooth muscle cells and A10 rat vascular smooth muscle cells, which were both successfully printed into contractile rings without any toxic effect of the nanoparticles (see Supplemental Fig. SF10 for graphs and Supplemental Table ST6 for p-values). Unlike traditional *ex vivo* vascular ring organ bath culture, this technique is compatible with cultured cells, reducing the dependence of researchers on live animals for intact tissues. Imaging and measurement of contraction within the rings were automated with the aid of a mobile device-based imaging system. Immunofluorescent staining suggested the presence of a contractile phenotype in the printed rings. These results demonstrate that the ring contraction seen in this assay is a measure of vasoactivity.

The main finding of this study was that bioprinted rings of vascular smooth muscle cells exhibited a concentration-dependent contractile response decoupled from toxicity. Known vasodilators, such as blebbistatin, forskolin, and verapamil reduced contraction (Fig. 2). Blebbistatin, which binds myosin light chain^30^, significantly reduced the rate of contraction. The decrease in pMLC stain intensity with higher concentrations of blebbistatin suggests that pMLC was reduced, which is consistent with its mechanism of action (Fig. 2). Gene expression profiling of ASMC rings exposed to 2.5 μM blebbistatin showed the significant upregulation of genes for inhibitors (PPP1R14A, ARHGEF1) of protein phosphatase activity, which could possibly reflect the promotion of contraction to compensate for the reduction (Supplemental Fig. SF11).

On the other hand, vasoconstrictors like norepinephrine, phenylephrine, and U46619 increased contraction (Fig. 3). Norepinephrine acts through adrenergic receptor signaling pathways to promote contraction^31^. There was a higher intensity pMLC stain in ASMC rings with norepinephrine, which is consistent with pMLC being a downstream pharmacodynamic marker of norepinephrine signaling. No overt changes were seen in pMLC staining intensity in A10 rings, which is consistent with the more modest effect of the drug on the rate of ring contraction (Fig. 3). A downregulation in caldesmon (CALD1) and protein phosphatase-1 (PPP1R12A) (Supplemental Fig. SF11) was found, possibly in response to the activation of these pathways as part of a negative feedback loop, as has been previously reported for RhoA-GEF activity in vascular smooth muscle cells in response to angiotensin II, a vasoconstrictor^32^. Overall, this study demonstrates that vascular ring contraction *in vitro* is related to biological markers of contractility, and thus can be used as a simple, yet robust endpoint for vasoactivity without the need for fresh tissue isolation from an animal.

The alterations in contractility detected in this assay were in general not a result of toxic responses by ASMC and A10 rings. Of the drug-cell type combinations tested, U46619 produced a toxic response in ASMC rings with higher concentrations (Supplemental Fig. SF2). As ASMC rings displayed increased contractility with higher concentrations (Fig. 4), the toxic effect of U46619 stands in contrast with previous reports of compound toxicity impairing ring closure in wound healing^24^. Moreover, a similar effect was not seen in A10 rings, which makes it unlikely that contraction was a result of toxicity. The general disconnect between toxicity and contractility in this assay demonstrates that altered contraction is not necessarily a measure of cytotoxicity.

The contractile responses were in agreement between the two cell types, with both cell types showing similarities in their responses to both vasoconstrictors and vasodilators, and the EC_50_’s for each cell type falling in a similar range (Table 1). One exception to this pattern was phenylephrine, which ASMCs were far less sensitive to (EC_50_ = 9.12 mM) than A10s (EC_50_ = 26.9 μM). To achieve these similar dose responses, however, ASMCs required 6 h of printing as opposed to 3 h of printing for A10s. The longer printing times allow cells to reorganize, align, and become contractile, as demonstrated by the immediate contraction of rings after adding drugs. This contractile phenotype was shown in this study by the general circumferential alignment of cells in the ring (Fig. 1) and the immediate contraction of rings (Supplemental Fig. SF1). Some compounds also exhibited different pharmacodynamic profiles between cell types, as exhibited by the timepoint at which contractile responses were measured (Supplemental Table ST1). The detection of these differences also highlight the ability of this assay to capture the contractile response over time as opposed to a single endpoint, and potentially as a means to detect changes in vasoactivity from a combination of drugs. Altogether, the amenability of the system to cells from both humans and preclinical species may enable *in vitro* assessment of inter-species sensitivities to pharmacological perturbation, a key facet of extrapolating from preclinical species used during drug discovery and development.

One key advantage of this assay is its higher throughput when compared to the single-vessel format typical of wire myography. Full plates (96 rings) can be printed simultaneously, with magnetic forces accelerating cell aggregation and ring formation. These rings can then be imaged as a whole plate using the mobile device, releasing constraints from expensive equipment and low-throughput imaging modalities^24^. As a result, an experiment could be performed from printing to analysis in less than 48 h. Throughput can be further expanded by adapting magnetic drive designs for higher well numbers, such as 384- and 1536-well plates in combination with automated imagers capable of achieving the necessary resolution. Overall, this ring assay was demonstrated to be conducive to high-throughput screening.

Another advantage of this assay is the ability to perform high-content screening using these rings without the need to isolate fresh tissue from a live animal. Ring contraction is a label-free endpoint, and is thus unhindered by diffusion limitations that come with reagent-based assays^33^. Moreover, the nanoparticles aid imaging with the mobile device by providing contrast, but do not interfere with any other assay, such as immunohistochemistry or Western blotting^15^,^19^,^20^,^22^,^25^. As a result, other tests can be performed on these rings post-contraction to explore mechanisms of action or contractile biology. The potential for high-content screening was demonstrated in this study by viability assays, immunohistochemical staining, and gene expression profiling of vascular smooth muscle ring to validate ring contraction as an endpoint of vasoactivity. Overall, this assay is advantageous for both high-throughput and high-content screening for vasoactivity.

Further development of this assay may include the introduction of vascular endothelial cells. In wire myography, endothelium is often denuded^7^,^8^,^9^, but endothelial cells are known to play a large role in regulating vascular tone^34^, and could affect results. Key considerations for the introduction of endothelial cells is the ratio and the method at which they are added to form a competent endothelium. Previous studies with valvular endothelial cells in co-culture suggest that when starting with a large population, they will form endothelium in 3D, but carry the risk of transdifferentiation into a mesenchymal phenotype^25^. The method of incorporating endothelial cells must also be considered, as they can either be mixed with vascular smooth muscle cells in the hopes that it would immediately reorganize to form a lumen, as has been shown previously with endothelial cells from white adipose tissue^19^, or use a more precise approach with different sized magnets to endothelial cells into a smaller ring within the vascular smooth muscle ring. The timing and competence of endothelial self-organization will determine how successful these methods will be. Future studies using this assay should explore the introduction of endothelial cells to add more relevance to this assay.

In conclusion, this study introduced an assay for vasoactivity using magnetic 3D bioprinting. These rings structurally and functionally mimic key facets of vasoactive blood vessel segments by contracting immediately after printing in response to vasoactive compounds. Immunohistochemistry and gene expression profiling revealed that the contraction properties of the magnetically printed rings were consistent with known vasoactive responses, without affecting cell viability. Furthermore, the results and methods used in this study demonstrate the adaptability of this assay to high-throughput and high-content screening, and may help overcome the limitations of existing *ex vivo* assessments of vascular contraction. This assay will aid in the reduction of animal use in labs studying vascular biology, a key tenant of the 3Rs principles (replacement, reduction, refinement), and allow safety labs focused on vasoactivity to become less reliant on animal models. Together, these results make the case for the use of magnetic 3D bioprinting to create representative environments *de novo* and improve *in vitro* vasoactivity screening.

## Methods

### Cell Culture

ASMCs (ScienCell, Carlsbad, CA) were cultured in specialized smooth muscle cell medium (ScienCell) containing 2% fetal bovine serum (FBS), 1% growth supplements, and 1% penicillin/streptomycin (P/S). ASMCs were cultured up to their fifth passage. A10s (ATCC, Manassas, CA) were cultured in Dulbecco’s Modified Eagle Medium (DMEM, ATCC) with 10% FBS (Gibco, Carlsbad, CA) and 1% P/S (Sigma-Aldrich). A10s were cultured up to their ninth passage. Cells were maintained in a humidified environment (37 °C, 5% CO_2_).

### Magnetic 3D Bioprinting

Cells were printed into rings as described previously in literature (Fig. 1a)^24^. At 70–80% confluence in 2D flasks, either ASMCs or A10s were magnetized by incubating the cells with a magnetic nanoparticle assembly (1 μL/1 × 10^4^ cells, NanoShuttle, Nano3D Biosciences, Houston, TX) overnight. The next day, cells were enzymatically detached off their substrate, counted, resuspended in media, then distributed into an ultra low-attachment 6-well plate (1.6 × 10^6^ cells/mL in 2 mL media, Corning, Tewksbury, MA). The plate was closed and a magnetic drive of 6 neodymium magnets was placed atop the plate to levitate the cells off the bottom and aggregate the cells, where the cells interacted with each other to form ECM. After 2 h of levitation, the structures were broken up with pipette action, resuspended in media and redistributed into a ultra low-attachment 96-well plate (2 × 10^5^ cells/ring in 270 μL media, Corning). This suspension of magnetized cells and ECM was then printed into rings by placing the plate on a magnetic drive of 96 ring-shaped neodymium magnets (4.76 mm OD × 1.59 mm ID). The magnets immediately attracted the cells to the bottom of the plate to form a 3D ring. The cells were left to print on the drive for either 3 h for A10s or 6 h for ASMCs. After printing, the plate was removed from the magnetic drive and allowed to rest for 5 min. The compounds were then added to each at 10 X the desired concentration (30 μL, n = 3 rings for 8 concentrations of each compound). The compounds in this study included (all purchased from Sigma-Aldrich, St. Louis, MO): vasodilators blebbistatin^30^, forskolin^35^, and verapamil;^36^ and vasoconstrictors norepinephrine^31^, phenylephrine^37^, and U46619^38^. Blebbistatin, verapamil, and U46619 were dissolved in 1% dimethylsulfoxide (DMSO), while forskolin, norepinephrine, and phenylephrine were dissolved in phosphate buffered saline (PBS). For each compound, negative controls were done with each experiment, and analyzed separate from other negative controls with the same solvent.

### Contraction Imaging and Analysis

After adding the drugs, ring contraction was captured using a mobile device-based imaging system as previously described^24^,^27^. Briefly, the plates were moved into an incubator and placed on top of an acrylic box with an iPod (32 GB iPod Touch, Apple Computer, Cupertino, CA) facing upwards. A light pad (LightPad A920, Artograph, Delano, MN) is placed atop the whole apparatus to illuminate the plate for imaging. The iPod is programmed using an application (Experimental Assistant, Nano3D Biosciences) to image the plate every 30 s for 24 h (n = 3 rings for 8 concentrations of each compound). After 24 h, the images were transferred to a separate computer, where image analysis was performed using custom code written in Python to measure ring area over time^24^,^27^. For each cell type and drug, a specific response was measured and compared to dosage (see Supplemental Table ST1 for the responses measured for each cell type and compound). Data points were rescaled between the maximum and minimum values. With this data, the dose response curves were fit to a sigmoidal curve (OriginPro, OriginLab, Northampton, MA), and the half maximum effective concentrations (EC_50_) were found from the fitted curve.

### Viability

Viability was assessed after contraction using a luminescent assay (CellTiter-Glo, Promega, Madison, WI). The plate of contracted rings was placed atop the magnetic drive to hold the rings down. Once the rings were held down, the media was removed and replaced with a luminescent detection reagent. After at least 15 min of incubation, the plate was read for luminescence on a plate reader. Data points were rescaled between the maximum and minimum values.

### Immunohistochemistry

Immunohistochemical staining was used to validate that ring contraction in this assay matched biological markers of contractility (n = 1 ring for 8 concentrations of each compound). Printed rings that were rested and allowed to contract for 5 min after drug exposure were fixed with 4% paraformaldehyde (Electron Microscopy Sciences, Hatfield, PA). Through this step and the remaining staining process, the plate was placed on the magnetic drive to hold the rings down while solutions were added and removed. The rings were fixed for at least 15 min, then washed with Tris buffered saline (TBS), and permeabilized with 0.2% Triton X-100 for 15 min. Once permeabilized, the rings were blocked using 1% donkey serum (Sigma-Aldrich) for 1 h. Next, the serum was replaced with the primary antibody solution, either for αSMA (1:100 in TBS, Abcam), or pMLC (1:200 in TBS, Abcam) to incubate overnight at 4 °C. The rings were then washed and stained using a fluorescent secondary antibody (1:200 in TBS, AlexaFluor, Invitrogen, Carlsbad, CA) for 1 h at room temperature, then counterstained using 4′,6-diamidino-2-phenylindole (1:1000 in TBS, DAPI, KPL, Gaithersburg, MD). Finally, the stained rings were imaged under both fluorescent and confocal microscopes.

### Statistical Analysis

One-way ANOVAs to determine the significance of the effect of compound concentration on ring contraction and viability were performed using analytical software (OriginPro). *Post hoc* Tukey’s tests were performed to compare groups. Significance was defined as *p* < 0.05. All data is presented as mean ± standard error.

## Additional Information

**How to cite this article**: Tseng, H. *et al.* A high-throughput *in vitro* ring assay for vasoactivity using magnetic 3D bioprinting. *Sci. Rep.* **6**, 30640; doi: 10.1038/srep30640 (2016).

## Supplementary Material

Supplementary Information

## Figures and Tables

**Table 1:** EC_50_’s found from the vascular ring assay using magnetically 3D bioprinted ASMCs and A10s.

Drug	EC_50_ (μM)	
	ASMC	A10
Blebbistatin	17.81	23.40
Forskolin	10.12	10.47
Verapamil	234.8	196.98
Norepinephrine	4.34	0.76
Phenylephrine	9.12 × 10^3^	26.9
U46619	4.68	3.46
