# Supplementary material for: A high-throughput in vitro ring assay for vasoactivity using magnetic 3D bioprinting
Source: Sci Rep. 2016 Aug 1;6:30640. doi: 10.1038/srep30640 (PMC4967891; doi:10.1038/srep30640)
Supplement: Supplementary Information [file srep30640-s1.doc]

**A high-throughput *in vitro* ring assay for vasoactivity using magnetic 3D bioprinting**

Hubert Tseng^1^, Jacob A. Gage^1^, William L. Haisler^1^, Shane K. Neeley^1^, Tsaiwei Shen^2^, Chris Hebel^2^, Herbert G. Barthlow^3^, Matthew Wagoner^3^, Glauco R. Souza^1,*^

**Supplemental Figures**


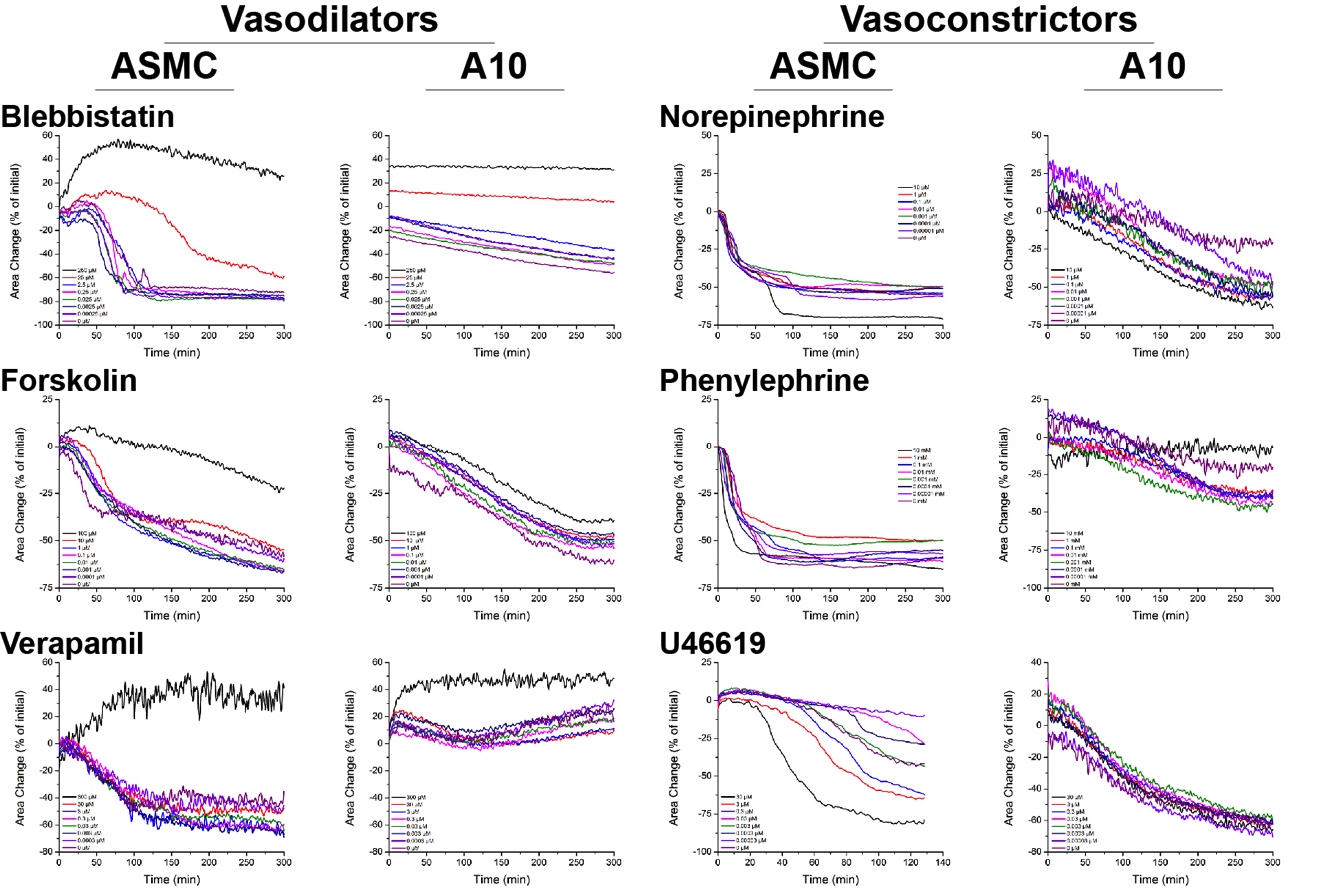


**Figure SF1**: *Time-dependent contraction of vascular smooth muscle rings.* ASMC and A10 ring contraction as a function of time and compound concentration. Note that in general, vasodilators had an expected dilatory effect on ASMC rings with higher compound concentrations, while vasoconstrictors had an expected contractile effect.


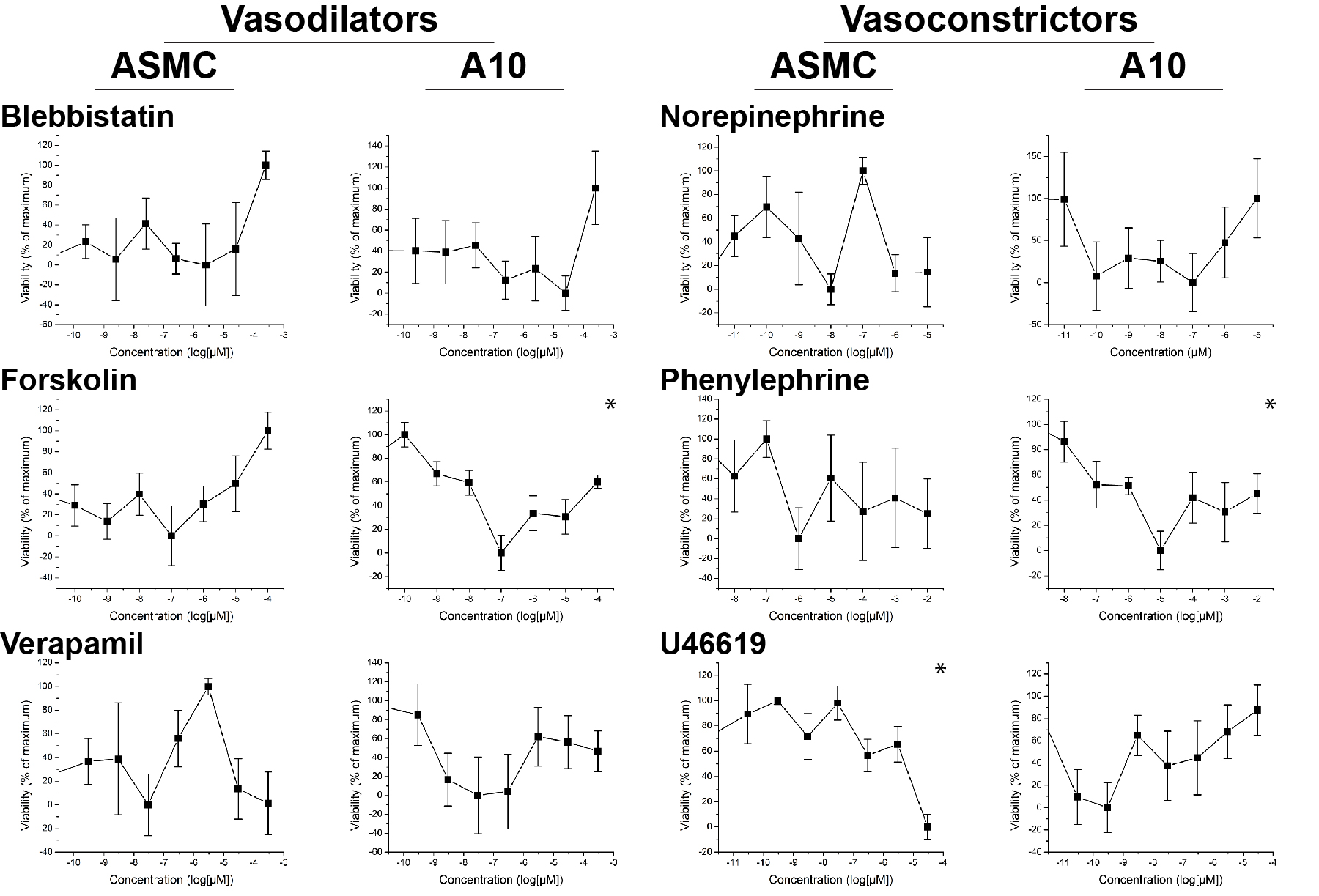


**Figure SF2**: *Viability of vascular smooth muscle rings*. Viability of ASMC and A10 rings as a function of compound concentration (n = 3 for 8 concentrations of each compound). Lines lead to control value on the left of the y-axis. In general, there was no significant compound effect on viability. The only toxic effect found was from U46619 on ASMC rings, which contrasts with previous reports. 100% = maximum viability, 0% = minimum viability. *: p < 0.05 effect of concentration.


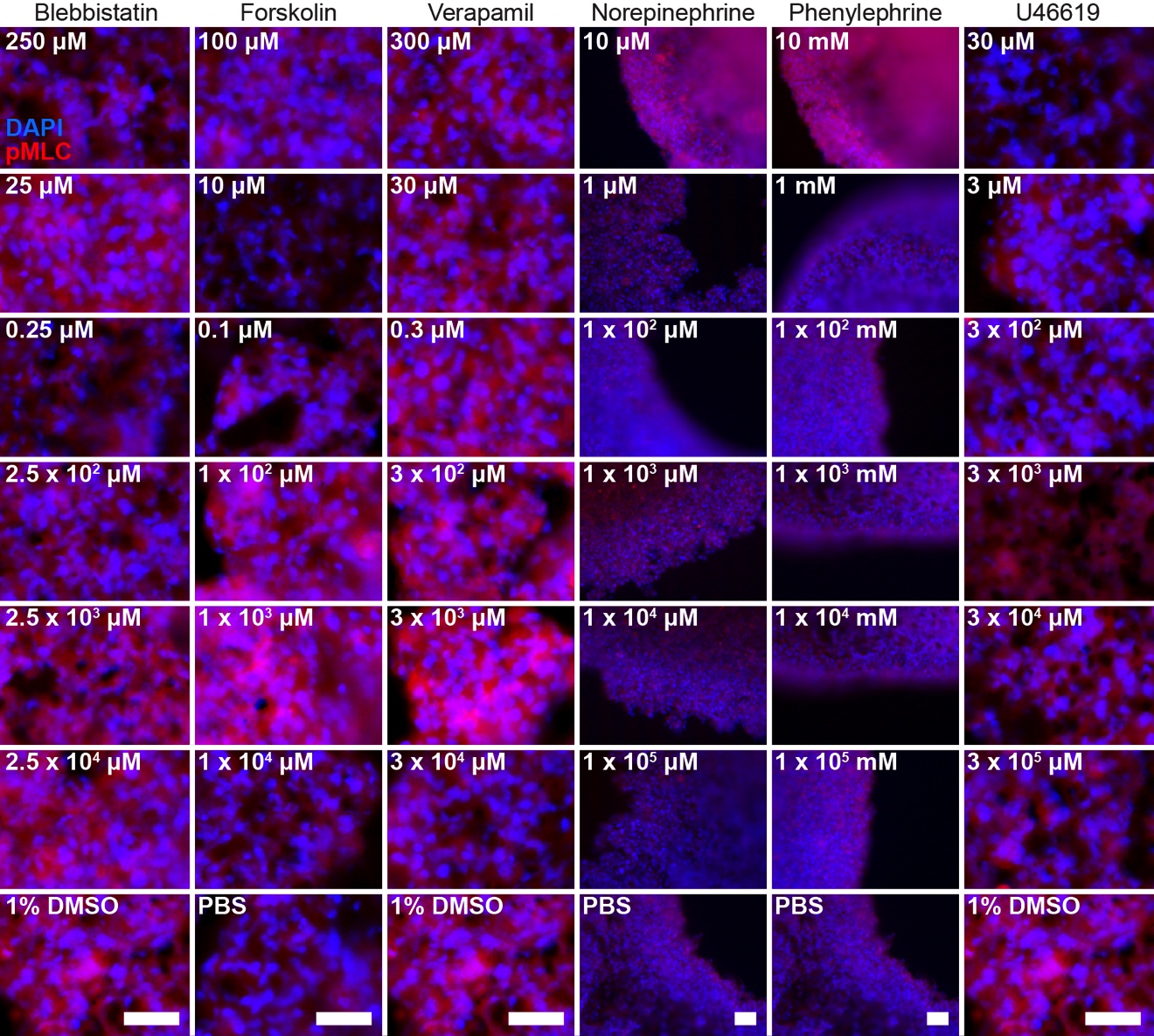


**Figure SF3**: *IHC stains of human primary aortic smooth muscle rings for pMLC.* IHC stains of ASMC rings for pMLC (red) for all compounds and concentrations after 5 min of contraction. Nuclei were counterstained with DAPI (blue). In general, for vasodilators (blebbistatin, forskolin, and verapamil), there was a slight reduction in pMLC stain intensity with increasing compound concentrations, particularly with blebbistatin and verapamil. For vasoconstrictors (norepinephrine, phenylephrine, and U46619), there was no observable difference in pMLC stain intensity. Scale bar = 50 μm.


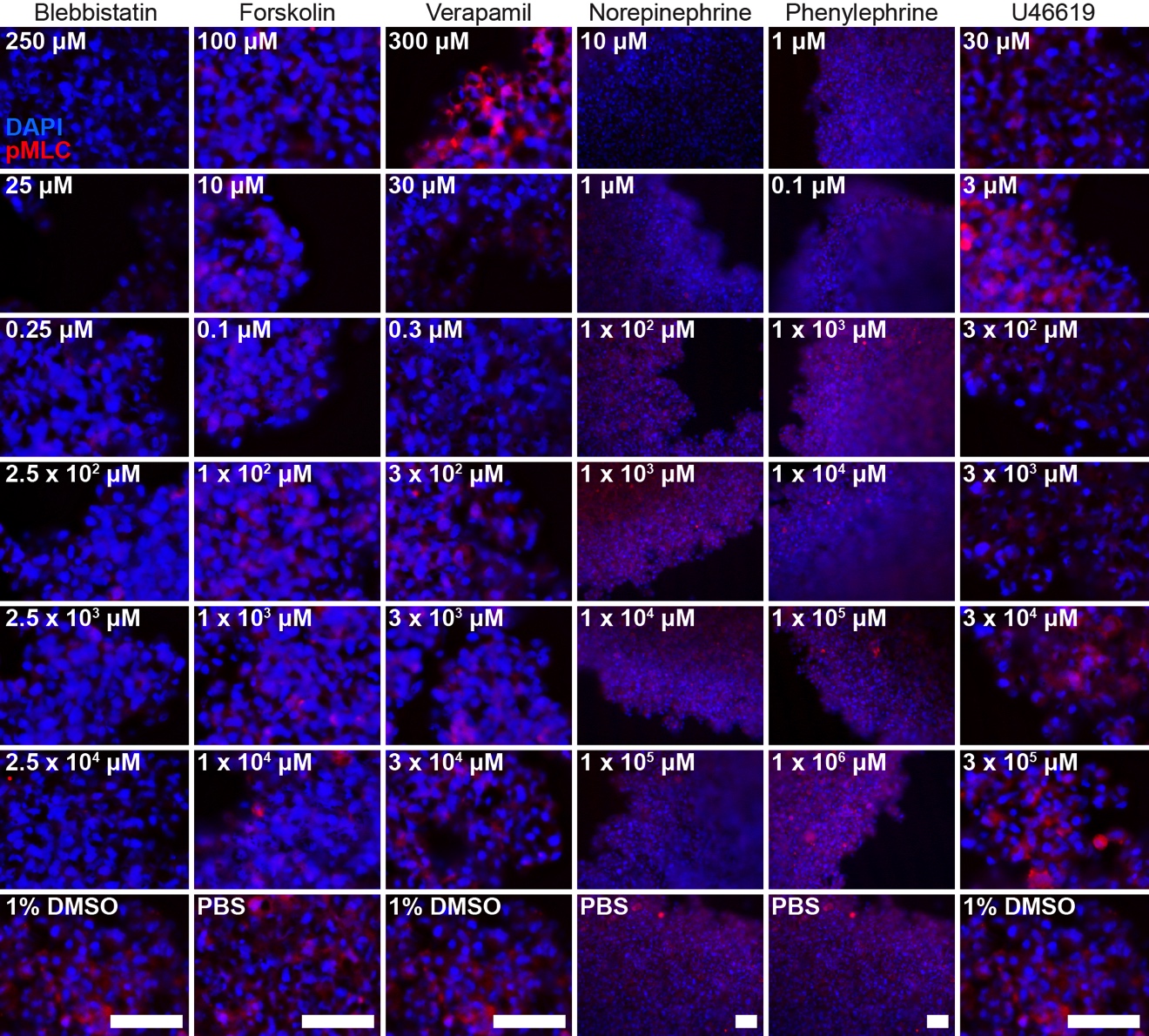


**Figure SF4**: *IHC stains of A10 vascular smooth muscle rings for pMLC.* IHC stains of A10 rings for pMLC (red) for all compounds and concentrations after 5 min of contraction. Nuclei were counterstained with DAPI (blue). In general, for vasodilators (blebbistatin, forskolin, and verapamil), there was a reduction in pMLC stain intensity with increasing compound concentrations. For vasoconstrictors (norepinephrine, phenylephrine, and U46619), there was no observable difference in pMLC stain intensity. Scale bar = 50 μm.


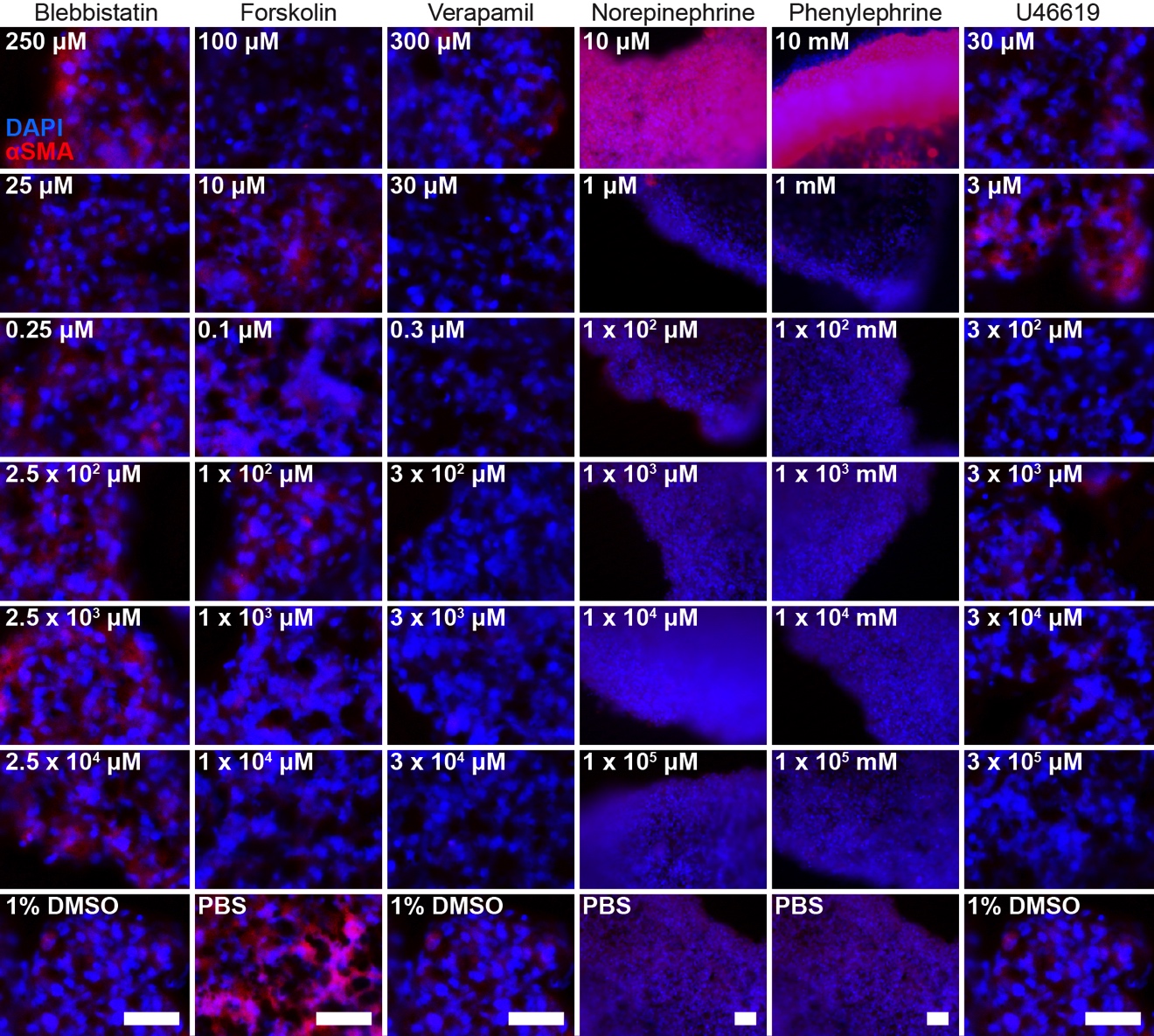


**Figure SF5**: *IHC stains of human primary aortic smooth muscle rings for αSMA.* IHC stains of ASMC rings for αSMA (red) for all compounds and concentrations after 5 min of contraction. Nuclei were counterstained with DAPI (blue). All compounds and concentrations confirmed the presence of αSMA and a contractile phenotype within the ring. Scale bar = 50 μm.


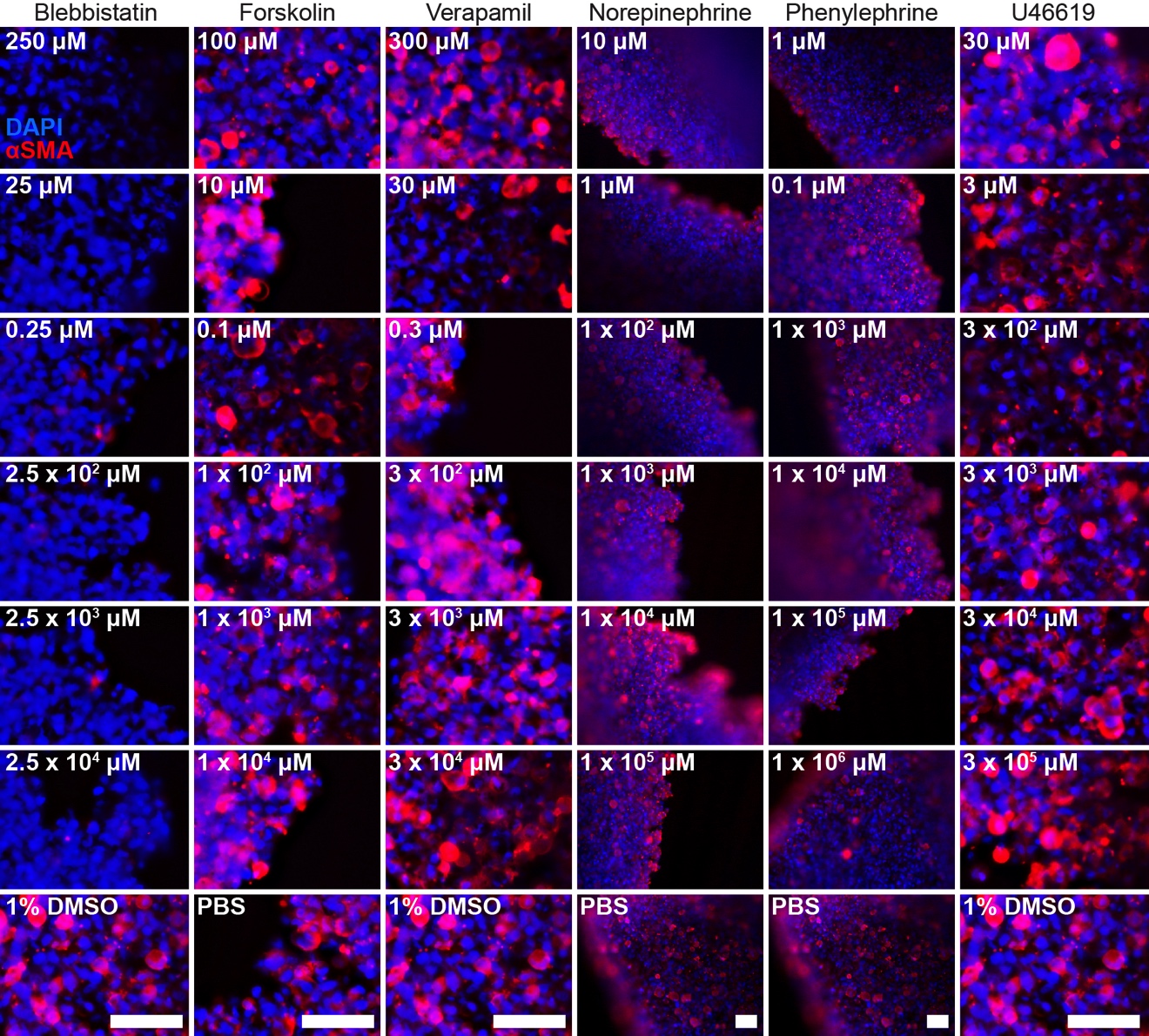


**Figure SF6**: *IHC stains of A10 vascular smooth muscle rings for αSMA.* IHC stains of A10 rings for αSMA (red) for all compounds and concentrations after 5 min of contraction. Nuclei were counterstained with DAPI (blue). All compounds and concentrations confirmed the presence of αSMA and a contractile phenotype within the ring. At all concentrations but control, A10 rings exposed to blebbistatin showed a starkly lower intensity stain compared to A10 rings exposed to other compounds. Scale bar = 50 μm.


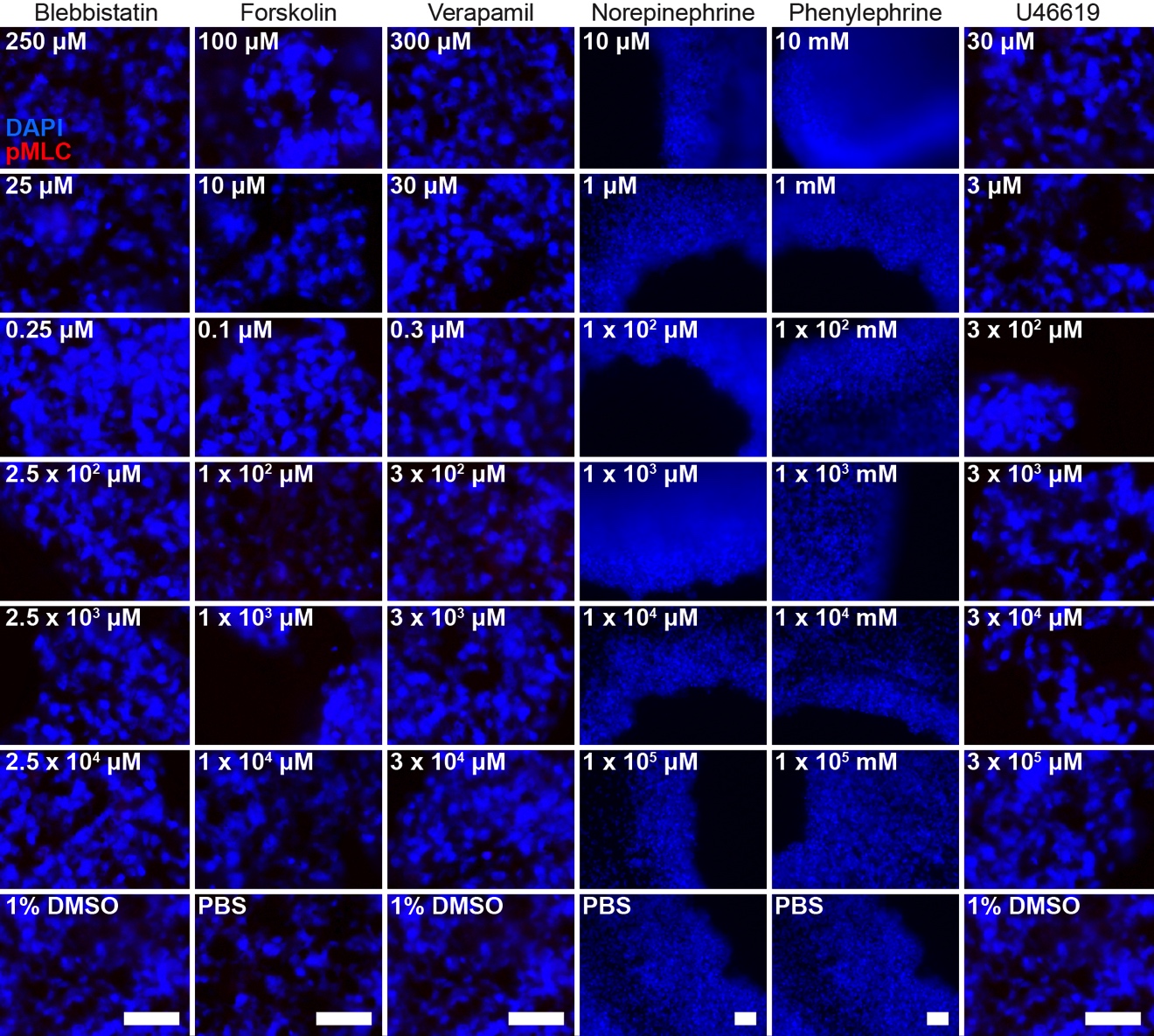


**Figure SF7**: *Negative controls for IHC stains of human primary aortic smooth muscle rings.* Negative controls for IHC stains of ASMC rings for αSMA and pMLC for all compounds and concentrations after 5 min contraction. Nuclei were counterstained with DAPI (blue). Scale bar = 50 μm.


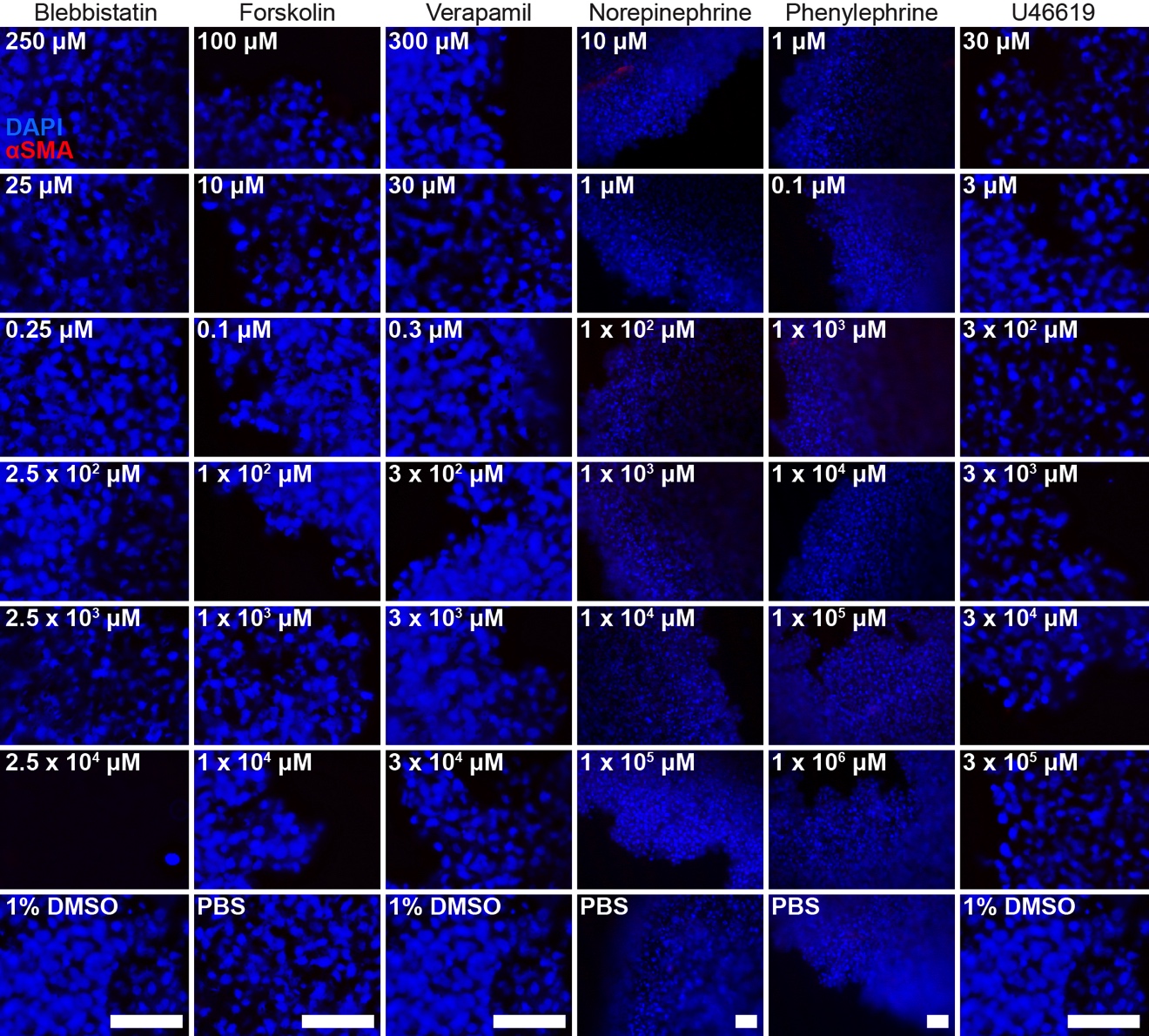


**Figure SF8**: *Negative controls for IHC stains of A10 vascular smooth muscle rings.* Negative controls for IHC stains of A10 rings for αSMA and pMLC for all compounds and concentrations after 5 min contraction. Nuclei were counterstained with DAPI (blue). Scale bar = 50 μm.


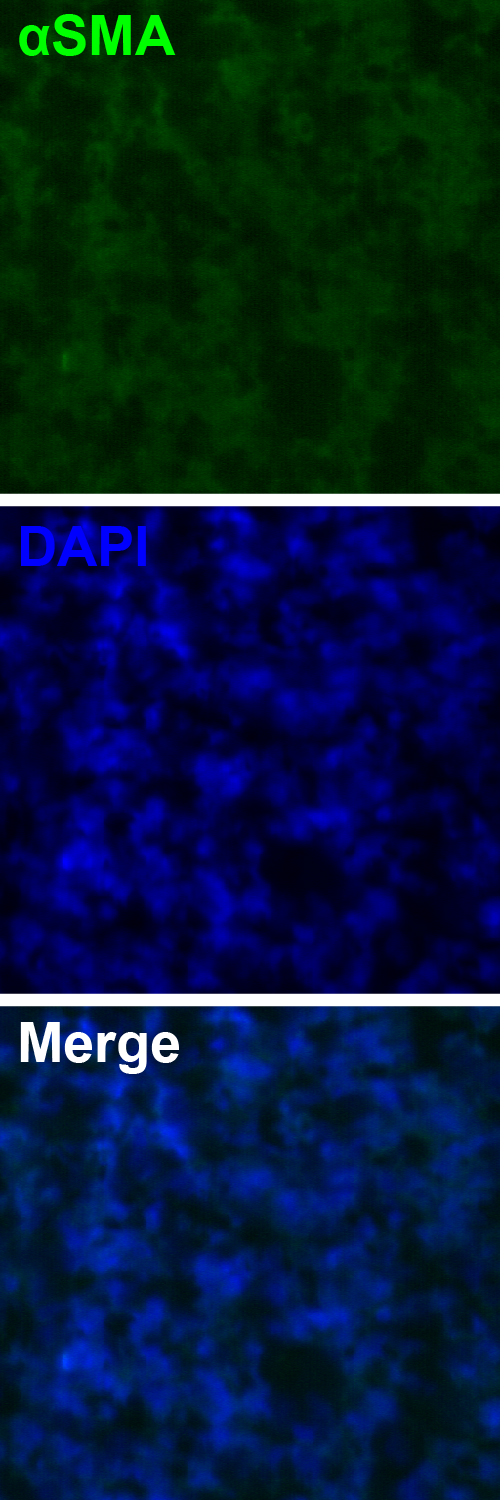


**Figure SF9**: *Negative controls for IHC stains of human primary aortic smooth muscle rings for* *αSMA*. Negative controls for immunohistochemical stain of an ASMC ring for αSMA (green) after 5 min contraction captured with confocal microscopy. Nuclei were counterstained with DAPI (blue). Scale bar = 50 μm.


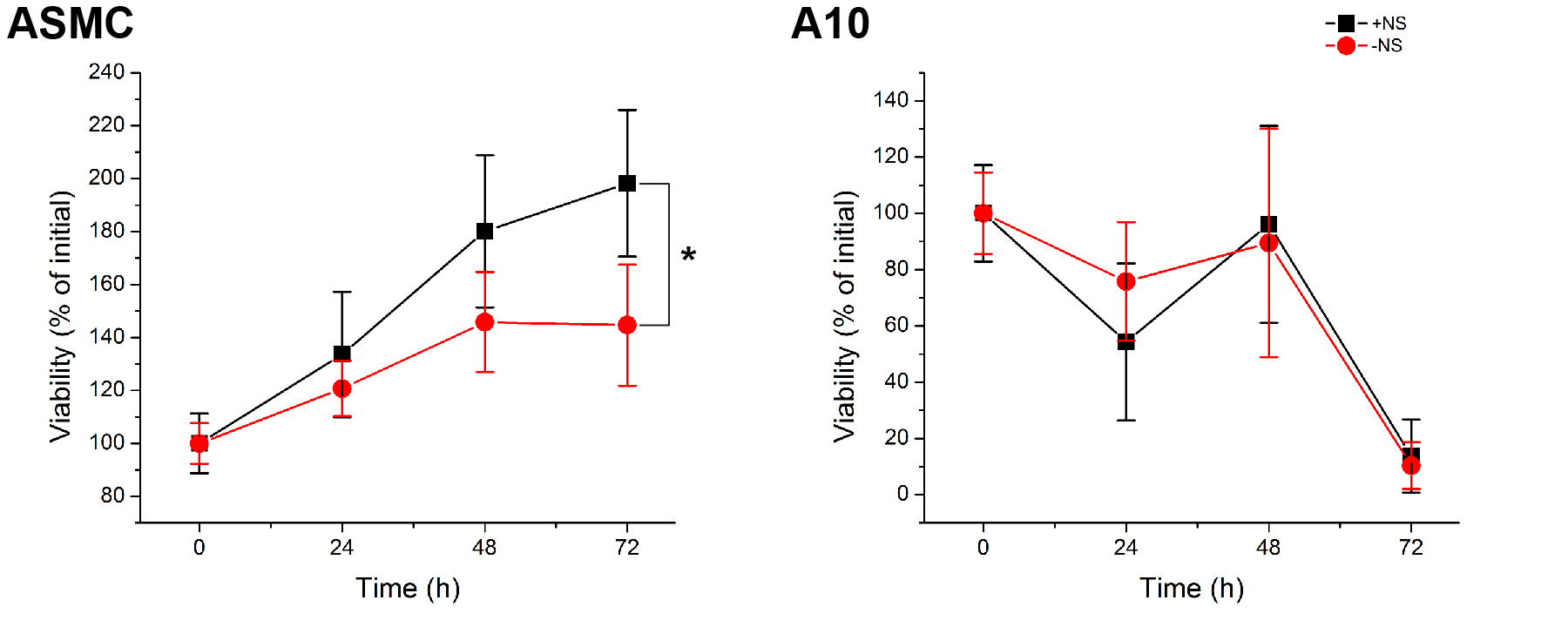


**Figure SF10**: *Effect of nanoparticles on viability*. Viability was measured in monolayers of ASMCs and A10s with (black) and without (red) NanoShuttle (NS). No effect was found on A10s, while ASMCs with NS actually grew faster than those without. *: *p* < 0.05 between groups.

**
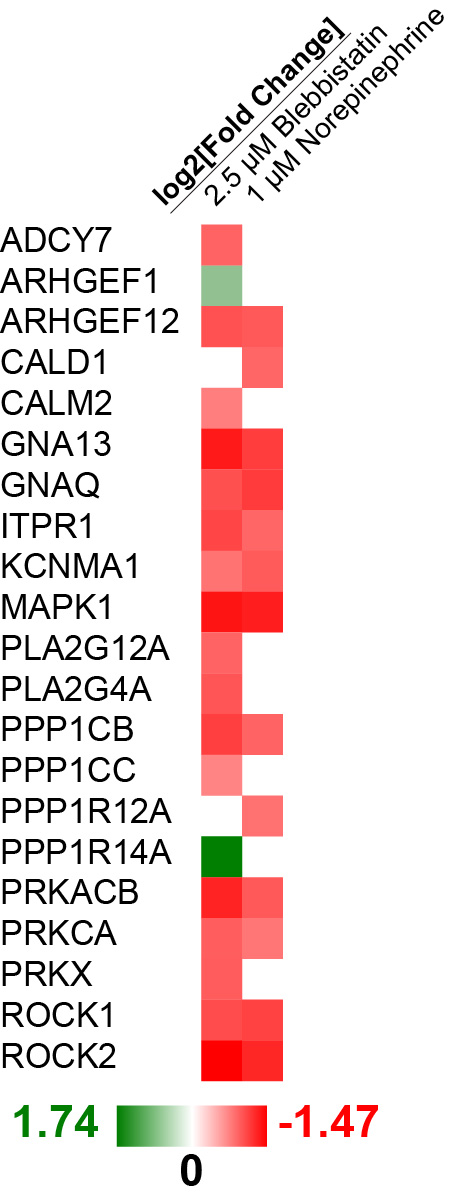
**

**Figure SF11**: *Effect of blebbistatin and norepinephrine on ASMC gene expression*. Heat map of genes in the vascular smooth muscle contraction pathway that were significantly affected by exposure of ASMC rings to either 2.5 μM blebbistatin or 1 μM norepinephrine (n = 10 rings per group). These concentrations were chosen as they were close to the measured EC_50_'s without risk of any toxic effect (Table 1). After contraction, the rings were snap frozen at -20ºC overnight and RNA was isolated (RNeasy Mini kit, Qiagen, Venlo, Netherlands). The RNA library was prepared (Illumina, San Diego, CA), with single-end reads of 50 nt were generated for each sample (HiSeq 200, Illumina), mapped to genomes (hg19 and rn4, Tophat v1.4.1) and assembled and analyzed with Cufflinks v2.2.1. When ASMC rings were exposed to blebbistatin, 8 genes were unique to blebbistatin, with 2 genes (PPP1R14A and ARHGEF1) upregulated, while all other genes were downregulated. When exposed to norepinephrine, ASMC rings had 2 unique genes downregulated. 11 genes were downregulated by both drugs, which likely reflects the dual roles of downstream markers masked by overall phenotypic changes. Decreases are shown in red, increases are shown in green, with darker intensities indicating a higher magnitude of change, and white indicating no significant difference.

**Supplemental Tables**

**Table ST1**: *Contraction endpoints.* Time over which contraction was measured for ASMC and A10 rings for each compound

| ***Drug*** | ***Response*** | |
| --- | --- | --- |
|  | ***ASMC*** | ***A10*** |
| Blebbistatin | 1 h | 1 h |
| Forskolin | 1 h | 2 h |
| Verapamil | 1 h | 2 h |
| Norepinephrine | 1.5 h | 4 h |
| Phenylephrine | 0.5 h | 0.5 h |
| U46619 | 1 h | 4 h |

**Table ST2**: *One-way ANOVA p-values*. p-values for effect of compound concentration on area change in ASMC and A10 rings

| ***Drug*** | ***p-value*** | |
| --- | --- | --- |
|  | ***ASMC*** | ***A10*** |
| Blebbistatin | 1.53 x 10^-4^ | 3.75 x 10^-5^ |
| Forskolin | 4.34 x 10^-5^ | 0.017 |
| Verapamil | 4.76 x 10^-5^ | 8 x 10^-3^ |
| Norepinephrine | 0.021 | 0.033 |
| Phenylephrine | 0.047 | 0.039 |
| U46619 | 4.57 x 10^-6^ | 0.043 |

**Table ST3**: *Tukey's testing of human primary aortic smooth muscle cell rings.* p-values from Tukey's testing of pairwise comparisons versus controls for each compound using ASMC rings

| Blebbistatin | | Forskolin | | Verapamil | | Norepinephrine | | Phenylephrine | | U46619 | |
| --- | --- | --- | --- | --- | --- | --- | --- | --- | --- | --- | --- |
| μM | *p* | μM | *p* | μM | *p* | μM | *p* | mM | *p* | μM | *p* |
| 250 | 4.8e-3 | 100 | 1.8e-5 | 300 | 4.9e-5 | 10 | 0.05 | 10 | 2.5e-3 | 30 | 2.4e-5 |
| 25 | 0.69 | 10 | 0.05 | 30 | 1 | 1 | 1 | 1 | 1 | 3 | 0.35 |
| 2.5 | 1 | 1 | 0.89 | 3 | 1 | 0.1 | 1 | 0.1 | 0.94 | 0.3 | 0.97 |
| 0.25 | 1 | 0.1 | 0.45 | 0.3 | 0.92 | 0.01 | 1 | 0.01 | 1 | 0.03 | 1 |
| 0.025 | 0.95 | 0.01 | 0.93 | 0.03 | 1 | 1e-3 | 0.93 | 1e-3 | 0.89 | 3e-3 | 1 |
| 2.5e-3 | 0.30 | 1e-3 | 0.77 | 3e-3 | 1 | 1e-4 | 1 | 1e-4 | 0.65 | 3e-4 | 1 |
| 2.5e-4 | 0.96 | 1e-4 | 0.37 | 3e-4 | 0.99 | 1e-5 | 1 | 1e-5 | 0.91 | 3e-5 | 1 |

**Table ST4**: *Tukey's testing of A10 vascular smooth muscle cell rings.* p-values from Tukey's testing of pairwise comparisons versus controls for each compound using A10 rings

| Blebbistatin | | Forskolin | | Verapamil | | Norepinephrine | | Phenylephrine | | U46619 | |
| --- | --- | --- | --- | --- | --- | --- | --- | --- | --- | --- | --- |
| μM | *p* | μM | *p* | μM | *p* | μM | *p* | mM | *p* | μM | *p* |
| 250 | 7.0e-5 | 100 | 0.05 | 300 | 0.05 | 10 | 0.02 | 10 | 0.05 | 30 | 0.05 |
| 25 | 0.04 | 10 | 0.73 | 30 | 0.97 | 1 | 0.05 | 1 | 0.41 | 3 | 1 |
| 2.5 | 0.88 | 1 | 0.76 | 3 | 1 | 0.1 | 0.03 | 0.1 | 0.84 | 0.3 | 1 |
| 0.25 | 1 | 0.1 | 1 | 0.3 | 1 | 0.01 | 0.15 | 0.01 | 0.53 | 0.03 | 1 |
| 0.025 | 1 | 0.01 | 1 | 0.03 | 1 | 1e-3 | 0.14 | 1e-3 | 0.44 | 3e-3 | 0.44 |
| 2.5e-3 | 0.95 | 1e-3 | 0.46 | 3e-3 | 1 | 1e-4 | 0.07 | 1e-4 | 0.99 | 3e-4 | 0.94 |
| 2.5e-4 | 0.82 | 1e-4 | 0.78 | 3e-4 | 1 | 1e-5 | 0.42 | 1e-5 | 1 | 3e-5 | 1 |

**Table ST5:** *Viability p-values.* p-values for the effect of concentration on viability within vascular smooth muscle rings

| ***Drug*** | ***p-value*** | |
| --- | --- | --- |
|  | ***ASMC*** | ***A10*** |
| Blebbistatin | 0.34 | 0.33 |
| Forskolin | 0.12 | 2.4 x 10^-4^ |
| Verapamil | 0.22 | 0.3 |
| Norepinephrine | 0.06 | 0.56 |
| Phenylephrine | 0.54 | 0.02 |
| U46619 | 0.02 | 0.08 |

**Table ST6:** *Effect of NS on viability*. p-value for the effect of NS on viability in monolayers after 72 h

| ***p-value*** | |
| --- | --- |
| ***ASMC*** | ***A10*** |
| 4.4 x 10^-3^ | 0.60 |
